# Novel Potential Probiotics from Chinese Baijiu Fermentation Grains: Dual Action of *Lactiplantibacillus plantarum* LTJ1/LTJ48 in Uric Acid Reduction and Gut Microbiota Restoration for Hyperuricemia Therapy in Mice

**DOI:** 10.3390/nu17132097

**Published:** 2025-06-24

**Authors:** Feiliang Zhong, Xiaomin Feng, Jun Cao, Miao Li, Jianxia Tian, Jiali Wang, Xuefang Wang, Xuegang Luo

**Affiliations:** Key Laboratory of Industrial Fermentation Microbiology of the Ministry of Education, College of Biotechnology, Tianjin University of Science and Technology, Tianjin 300457, China; flzhong91@tust.edu.cn (F.Z.); fxmxka@mail.tust.edu.cn (X.F.); caojun@mail.tust.edu.cn (J.C.); lm23815001@mail.tust.edu.cn (M.L.); tian_tjx@mail.tust.edu.cn (J.T.); wangjiali816@163.com (J.W.); wangxuefang@mail.tust.edu.cn (X.W.)

**Keywords:** hyperuricemia, *Lactiplantibacillus plantarum* LTJ1 and LTJ48, UA metabolism, gut microbiota dysbiosis, Chinese baijiu fermenting grains

## Abstract

Objectives: Hyperuricemia (HUA) is a metabolic disorder linked to serious complications, yet current treatments face safety limitations. This study aimed to identify novel probiotic strains from Chinese Baijiu fermentation grains with dual-action mechanisms for HUA management—direct uric acid (UA) reduction and gut microbiota restoration. Methods: Two Lactiplantibacillus plantarum strains (LTJ1/LTJ48) were screened for purine/nucleoside degradation using HPLC. Their efficacy was evaluated in HepG2 cells and HUA mice. Key assessments included UA levels, renal/hepatic markers (AST, CRE, BUN), ADA/XOD activity, UA transporter expression (URAT1, GLUT9, ABCG2), and 16S rRNA-based microbiota analysis. Results: LTJ1/LTJ48 degraded >97% of purines/nucleosides in vitro. In HUA mice, they reduced serum UA by 31.0% (LTJ1) and 51.5% (LTJ48), improved renal/hepatic function, and suppressed ADA activity. They modulated UA transporters and restored gut microbiota. Conclusions: LTJ1/LTJ48 exhibit multi-target HUA alleviation via purine degradation, ADA inhibition, UA transporter regulation, and microbiota remodeling, offering a safer probiotic-based alternative to conventional therapies. Their translational potential warrants further clinical exploration.

## 1. Introduction

Hyperuricemia (HUA), defined as a serum uric acid (UA) level exceeding 420 μmol/L on two consecutive measurements, results from purine metabolism dysregulation and/or renal-intestinal excretion impairment [1,2]. This condition is clinically linked to multi-organ complications such as cardiovascular diseases [3], chronic kidney disease [4], and metabolic syndrome [5], with its global prevalence rising markedly in recent decades, especially in younger cohorts. Pathogenetically, HUA originates from two distinct mechanisms: disordered purine metabolism causing UA overproduction, and underexcretion primarily through renal or intestinal pathways [6]. Sustained HUA induces urate crystal deposition in joints and soft tissues, triggering subsequent sterile inflammation and gout onset, with approximately 20% of cases progressing to clinical gout [7]. Critically, emerging evidence identifies the gut–kidney axis as a master regulator of systemic urate homeostasis, establishing the intestine as both an immunometabolic interface and an alternative compensatory excretion route [8].

Current therapeutic strategies for HUA management integrate pharmacotherapy with nutritional approaches [9,10]. Clinically endorsed agents comprise three mechanistic classes: xanthine oxidase (XOD) inhibitors (such as allopurinol and febuxostat) that suppress de novo urate synthesis through competitive inhibition of hypoxanthine oxidation [11]; uricosuric drugs (predominantly benzbromarone) targeting urate transporter 1 (URAT1) to enhance renal clearance [12]; and recombinant uricolytic enzymes (notably rasburicase) mediating the enzymatic conversion of UA to water-soluble allantoin [13]. Nevertheless, significant safety concerns constrain their application. Allopurinol carries risks of severe cutaneous adverse reactions [14], febuxostat demonstrates elevated cardiovascular event rates [15], and benzbromarone potentiates drug-induced liver injury [16], whereas probenecid–aspirin polytherapy compromises both urate-lowering efficacy and antimicrobial safety profiles [17,18]. These limitations underscore the urgent need for safer alternatives.

Accumulating evidence positions gut microbiota dysbiosis as a pathogenic driver of HUA [19]. High-purine diets exert dual pathological effects: inducing structural damage to the intestinal barrier and triggering taxonomic shifts in microbial communities—mechanistically linked to impaired UA excretion and breakdown of immune tolerance [19,20,21]. Notably, purine-metabolizing probiotics derived from traditional fermented foods have garnered research attention as multi-mechanistic therapeutic candidates, exhibiting enteric purine sequestration, microbiota restitution, and immunometabolic modulation capabilities [22,23]. Of particular interest, *Lactiplantibacillus plantarum* (*L. plantarum*)—a phylogenetically diverse species enriched in fermented substrates—has demonstrated translational potential in metabolic disorders through xanthine oxidoreductase activity and microbiota-derived metabolite production [24,25].

In order to develop and explore the ability of potential probiotics to improve hyperuricemia, in this study, we identified and characterized two *L. plantarum* strains (designated LTJ1 and LTJ48) from Chinese baijiu fermentation grains. We hypothesized their therapeutic potential for HUA intervention through three mechanistic axes: purine/nucleoside degradation capacity, urate-lowering efficacy in cellular and murine models, and microbiome-modulating metabolic regulation of UA homeostasis.

## 2. Materials and Methods

### 2.1. In Vitro Screening of Lactiplantibacillus with High Purine and Nucleoside Degradation Capability

*Lactiplantibacillus* strains were previously isolated and screened from the fermented grains of soy sauce-flavored baijiu produced in northern China. Primary cultures were established by inoculating single colonies into 1 mL sterile MRS broth (AOBOXING), followed by 12 h incubation at 37 °C. Secondary cultures were prepared through 2% (*v*/*v*) inoculation of fresh MRS medium with primary culture under identical incubation conditions. Following centrifugation at 4000× *g* for 3 min, bacterial pellets from secondary cultures underwent triple washing with sterile saline. Washed cell suspensions were then incubated at 37 °C for 8 h in the prepared 0.2 g/L of inosine and adenosine (Meilunbio, Dalian, China) or 0.2 g/L of guanine and hypoxanthine (Macklin, Shanghai, China) mixture buffer. Reactions were terminated with 0.1 mol/L HClO_4_ (Century Aokebio, Wuhan, China).

Subsequent HPLC analysis (20 μL injection volume) employed a Tri-Sal Plus C18 column (SALA2-5046250, Agilent Technologies, Santa Clara, CA, USA) with the following parameters: Mobile phase: 20 mmol/L KH_2_PO_4_ (pH 3.0, Jiangtian Chemical, Nantong, China) containing 10% methanol (Concord Technology, Hong Kong, China), Flow rate: 1 mL/min, Column temperature: 25 °C, Detection: 254 nm (hypoxanthine, inosine, adenosine); 273 nm (guanine). Quantification was performed using external calibration curves established for each analyte. Substrate degradation rates were calculated based on peak area reduction compared to abiotic controls.

### 2.2. Strain Identification

Genomic DNA of *L. plantarum* strains were isolated using the Tianamp Bacteria DNA Kit. A 16S rRNA gene amplification was performed through PCR with universal primers 27F (5′-AGAGTTTGATCCTGGCTCAG-3′) and 1492R (5′-GGTTACCTTGTTACGACTT-3′). The PCR reaction mixture (25 μL total volume) contained 17.0 μL sterile ddH_2_O, 1.0 μL each of forward and reverse primers (10 μmol/L), 1.0 μL dNTPs (2.5 mmol/L), 0.5 μL Taq DNA polymerase (5 U/μL), 2.5 μL 10× PCR buffer, and 2.0 μL DNA template. Thermal cycling parameters consisted of an initial denaturation at 95 °C for 5 min, followed by 30 cycles of denaturation (95 °C, 45 s), annealing (56 °C, 1 min), and extension (72 °C, 1 min), with a final extension at 72 °C for 10 min. PCR products were purified and subjected to bidirectional sequencing through a commercial sequencing service (Genewiz, Suzhou, China). Sequence alignment and species identification were performed using the BLASTn algorithm against the NCBI GenBank database (https://blast.ncbi.nlm.nih.gov, accessed on 10 April 2023).

### 2.3. Growth Kinetics Determination

Bacterial growth kinetics were monitored through optical density measurements at 600 nm (OD_600_) using a spectrophotometric method. The strain was inoculated into MRS broth at 2% (*v*/*v*) inoculum size and incubated at 37 °C for 36 h. To establish the growth curve, aliquots were aseptically sampled at 4 h intervals for OD600 determination and plotting cultivation time against corresponding OD600 values.

### 2.4. Induction of HUA Cell Model

HepG2 cells were maintained in DMEM (Gibco, Carlsbad, CA, USA) supplemented with 10% fetal bovine serum (HyClone, Logan, UT, USA) under 5% CO_2_ at 37 °C. At 80–90% confluence during the logarithmic growth phase, cells were trypsinized and seeded in 6-well plates at a density of 1 × 10^6^ cells/well. Following a 24 h adhesion period, cultures were washed twice with PBS (pH 7.4) before experimental interventions. For HUA induction, three experimental groups were established: NC group: received 2 mL fresh basal medium; model group: treated with 2 mL PBS-supplemented medium; and treatment group: exposed to 15% (*v*/*v*) filter-sterilized *L. plantarum* metabolites in complete medium. After 24 h pre-treatment, all groups (except NC) received adenosine solution (0.25 mmol/L final concentration) for purine nucleotide loading. Following subsequent 24 h incubation, xanthine oxidase (XOD, Nanjing Jiancheng, Nanjing, China) was introduced at 0.005 U/mL concentration to initiate UA biosynthesis. The enzymatic reaction was terminated after 12 h by immediate supernatant collection. The dosage and time used were verified by a CCK-8 experiment.

### 2.5. Quantitative UA Analysis

The supernatant from the wells was collected. UA concentrations were determined using a commercial enzymatic colorimetric assay kit (Nanjing Jiancheng) following the manufacturer’s protocol. Absorbance measurements at 293 nm were performed in triplicate using a microplate reader, with UA standards establishing the calibration curve. Data normalization was performed against total cellular protein content quantified by a BCA assay.

### 2.6. Animal Experiment Design

All experimental protocols were approved by the Animal Ethics Committee of Tianjin University of Science and Technology and conducted in compliance with institutional guidelines for laboratory animal care. Fifty male SPF Kunming mice (4 weeks old, 18–22 g) purchased from SPF Biotechnology Co., Ltd. (Beijing, China) were acclimatized for 7 days under controlled conditions (24 ± 2 °C, 52 ± 5% humidity, 12 h light/dark cycle).

Mice were randomly divided into five groups (n = 10/group): negative control (NC); HUA model (M); LTJ1 intervention (1 × 10^8^ CFU *L. plantarum* LTJ1); LTJ48 intervention (1 × 10^8^ CFU *L. plantarum* LTJ48); and allopurinol control (AP). HUA was induced via daily oral gavage of 10 g/kg yeast extract combined with intraperitoneal injection of 300 mg/kg potassium oxonate (all groups except NC). Intervention groups received corresponding bacterial solutions 1 h post-modeling. Treatments continued for 21 d with daily dose adjustment based on body weight. After final administration, blood samples were collected via orbital puncture. Mice were then euthanized by cervical dislocation for tissue harvesting (liver, kidney, small intestine, colon/cecum contents), and the tissues were stored at −80 °C for subsequent analyses.

### 2.7. RT-qPCR Analysis

Total RNA was isolated from cellular samples and murine tissues (kidney/small intestine) using Trizol reagent (Sigma, St. Louis, MO, USA) following standard extraction protocol. RNA integrity was confirmed by spectrophotometry prior to reverse transcription. cDNA was synthesized using the GoScript™ Reverse Transcription System (Promega, Fitchburg, WI, USA) according to the manufacturer’s instructions. qPCR was performed using SYBR^®^ Green Master Mix (DBI^®^ Bioscience, Shanghai, China) on a StepOnePlus™ Real-Time PCR System. Each 20 μL reaction contained 10 μL 2 × SYBR Green Mix, 0.4 μL ROX passive reference dye, 1 μL gene-specific primers (10 μM), 1 μL cDNA template, and 7.6 μL nuclease-free water (Solarbio, Beijing, China). Reactions were performed in triplicate with the following thermal profile: 95 °C for 10 min, 40 cycles of 95 °C (15 s), 60 °C (30 s), and 72 °C (30 s). Gene expression quantification was calculated using the 2^−ΔΔCT^ method normalized to GAPDH as an endogenous control. Primer sequences are listed in Table 1.

### 2.8. DNA Extraction and 16S rRNA Gene Amplification

Mouse colonic contents were homogenized and subjected to genomic DNA extraction using a commercial kit (Solarbio, Beijing, China) following the manufacturer’s protocol. The hypervariable V3–V4 region of the bacterial 16S rRNA gene was amplified using primers 338F (5′-ACTCCTACGGGAGGCAGCAG-3′) and 806R (5′-GGACTACHVGGGTWTCTAAT-3′). PCR reactions (25 μL) contained Fast Pfu DNA polymerase (TransGen, Beijing, China) and were performed under the following conditions: 95 °C for 3 min; 35 cycles of 95 °C (30 s), 55 °C (30 s), and 72 °C (45 s); and final extension at 72 °C for 10 min. Amplicons were verified by 1.0% agarose gel electrophoresis, purified using a gel extraction kit, and subjected to a second round of PCR for index addition. Sequencing libraries were constructed by ligating Illumina adapters and quantified by Qubit fluorometry. Paired-end sequencing (2 × 250 bp) was performed on an Illumina HiSeq 2500 platform. Raw sequencing data were processed using bioinformatic pipelines (QIIME2 and DADA2) for quality filtering, ASV clustering, and taxonomic classification against the SILVA database to characterize microbial community composition and diversity.

### 2.9. Statistical Analysis

All statistical analyses were performed using GraphPad Prism 8 software. For comparisons between two independent groups, two-tailed Student’s *t*-tests were applied to assess statistical significance. The significance thresholds were defined as follows: not significant (ns) for *p* > 0.05; * *p* < 0.05; ** *p* < 0.01; *** *p* < 0.001. Quantitative data are presented as mean ± standard deviation (SD) from three independent biological replicates.

## 3. Results

### 3.1. Solation and Characterization of Nucleoside and Purine-Degrading L. plantarum LTJ1 and LTJ48

**Figure 1:**
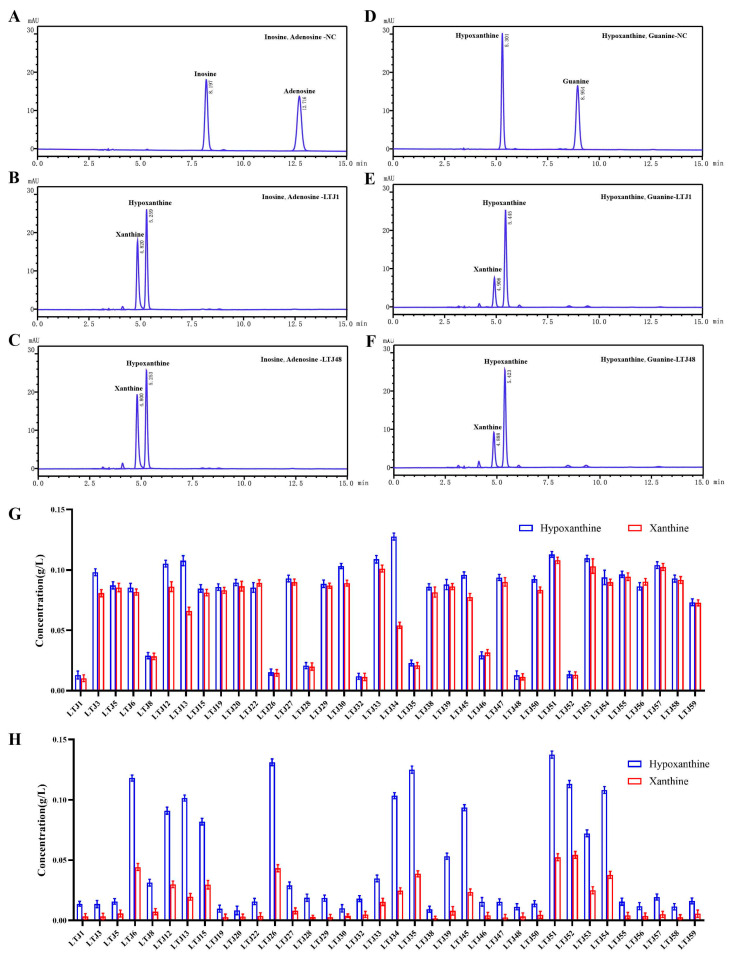
HPLC analysis of nucleoside and purine degradation by L. plantarum LTJ1 and LTJ48. (**A**) Chromatogram of nucleoside mixture (0.2 g/L adenosine and 0.2 g/L inosine); (**B**) degradation profiles of adenosine and inosine by LTJ1 and LTJ48; (**C**,**D**) chromatogram of purine mixture (0.2 g/L guanine and 0.2 g/L hypoxanthine); (**E**) degradation profiles of guanine and hypoxanthine by LTJ1 and LTJ48; and (**F**) screening results of 36 Lactobacillus strains for nucleoside (**G**) and purine (**H**) degradation. Characteristic retention times for key metabolites: inosine (8.197 min), adenosine (12.716 min), guanine (8.954 min), hypoxanthine (5.301 min), and xanthine (4.820 min).

To identify bacterial candidates with nucleoside and purine-degrading capabilities, 36 *Lactobacillus* strains were rigorously screened by incubating them for 8 h in PBS containing a nucleoside or purine mixture (0.2 g/L). Metabolic profiles were analyzed via HPLC, with characteristic absorption peaks identified for key substrates: inosine (8.197 min), adenosine (12.716 min), guanine (8.954 min), hypoxanthine (5.301 min), and xanthine (4.820 min) (Figure 1A–F). HPLC analysis revealed a consistent metabolic pattern: Enzymatic conversion by *Lactobacillus* strains generated xanthine and hypoxanthine as terminal products, accompanied by substrate depletion. Among all candidates, *L. plantarum* LTJ1 and LTJ48—previously isolated from Chinese baijiu fermentation grains—exhibited exceptional degradation efficiency (Figure 1G,H). LTJ1 completely degraded adenosine (100%) and inosine (99.5%), with 98.01% guanine and 12.0% hypoxanthine degradation, yielding a total purine reduction of 35.2% within 8 h (Figure 1B,E,G,H). Similarly, LTJ48 degraded 100% of adenosine and 99.2% of inosine, along with 97.7% of guanine and 27.6% of hypoxanthine, achieving a total purine degradation rate of 42.2% within 8 h (Figure 1C,F–H). These findings suggest that *L. plantarum* LTJ1 and LTJ48 may serve as promising candidates for the intervention of HUA.

### 3.2. Molecular Identification and Growth Characterization of L. plantarum LTJ1 and LTJ48

**Figure 2:**
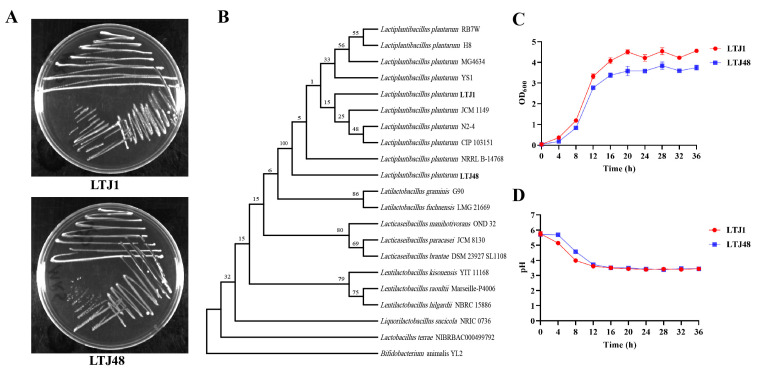
Molecular and phenotypic characterization of *L. plantarum* LTJ1 and LTJ48. (**A**) Colony morphology on MRS agar after 48 h incubation (37 °C); (**B**) phylogenetic tree reconstructed by neighbor-joining method based on 16S rRNA gene sequences; (**C**) the growth curve of LTJ1 and LTJ48; and (**D**) the acid production capacity of LTJ1 and LTJ48.

Following isolation and purification via three-zone streaking, both LTJ1 and LTJ48 formed opaque, white circular colonies on agar plates (Figure 2A). Growth curve analysis revealed comparable proliferation patterns, with both strains entering the stationary phase after 20 h of cultivation (Figure 2B). Molecular identification was performed by amplifying 16S rRNA genes through PCR, followed by sequencing and comparative analysis against the GenBank bacterial database. Phylogenetic reconstruction (neighbor-joining method) demonstrated 97.0% (LTJ1) and 98% (LTJ48) sequence similarity to *L. plantarum* type strains, confirming their taxonomic classification (Figure 2C). The strains have been deposited in the China General Microbiological Culture Collection Center (CGMCC) under accession numbers CGMCC No. 25123 (LTJ1) and CGMCC No. 25124 (LTJ48).

### 3.3. L. plantarum LTJ1 and LTJ48 Suppress UA Biosynthesis in HUA HepG2 Cells

**Figure 3:**
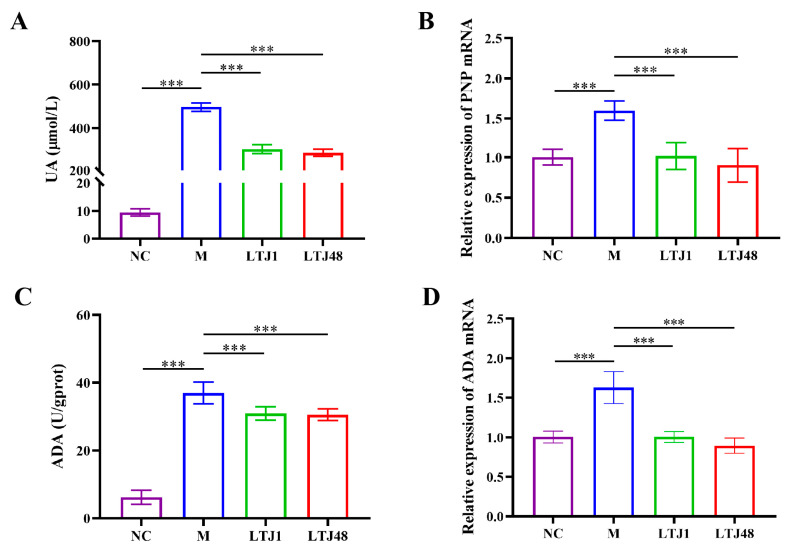
*L. plantarum* LTJ1 and LTJ48 attenuate HUA in HepG2 hepatocytes. (**A**) Intracellular UA levels after 24 h co-culture; (**B**) RT-qPCR analysis of PNP mRNA expression; (**C**) ADA enzymatic activity; and (**D**) ADA gene expression quantified by RT-qPCR. NC was the control group. M was the HUA cell model group. The LTJ1 group is the postbiotics of *L. plantarum* LTJ1 (15% *v*/*v*). The LTJ48 group is the postbiotics of *L. plantarum* LTJ48 (15% *v*/*v*). Data expressed as mean ± SD (n = 4 biological replicates), *** *p* < 0.001.

To evaluate the anti-HUA potential of LTJ1 and LTJ48 metabolites (postbiotics), we established a HUA cell model using HepG2 hepatocytes. Treatment with 15% (*v*/*v*) postbiotics from LTJ1 and LTJ48 significantly reduced intracellular UA levels by 40.0% (*p* < 0.001) and 43.6% (*p* < 0.001), respectively, compared to untreated HUA controls (Figure 3A). We further investigated its mechanistic basis through multi-omics analysis. Targeted enzymatic assays revealed marked suppression of purine nucleoside phosphorylase (PNP) activity and adenosine deaminase (ADA) activity (*p* < 0.001 vs. model group), paralleled by downregulation of PNP and ADA gene expression (Figure 3B–D).

### 3.4. L. plantarum LTJ1 and LTJ48 Ameliorate HUA in Mice Through ADA Suppression

**Figure 4:**
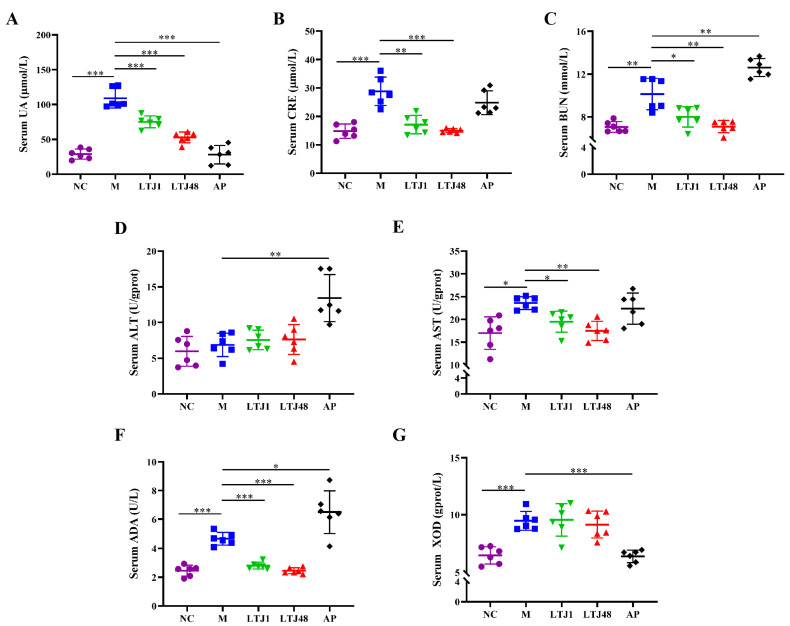
Influence of *L. plantarum* LTJ1 and LTJ48 on physiological and biochemical indices of HUA mice. (**A**) Serum UA levels; (**B**) serum CRE levels; (**C**) serum BUN levels; (**D**) serum ALT levels; (**E**) serum AST levels; (**F**) serum AST levels; and (**G**) serum XOD levels. NC was the control group. M was the HUA model group. AP was the group treated with the positive drug allopurinol. The LTJ1 group was given live *L. plantarum* LTJ1. The LTJ48 group was given inactivate *L. plantarum* LTJ48. Data represent mean ± SD (n = 6/group). Statistical significance vs. HUA model group: * *p* < 0.05, ** *p* < 0.01, *** *p* < 0.001.

To validate the anti-HUA effects in vivo, HUA mouse model was established through daily administration of yeast extract and potassium oxonate for 21 days, simulating a long-term high-purine dietary intake. Mice were orally supplemented with *L. plantarum* LTJ1 or LTJ48 (1 × 10^9^ CFU/day). Serum analysis demonstrated successful model induction, with the HUA group exhibiting a 3.8-fold increase in UA compared to controls (*p* < 0.001) (Figure 4A). Both strains significantly reduced serum UA levels: LTJ1 by 31.0% and LTJ48 by 51.5% (*p* < 0.001 vs. HUA group) (Figure 4A). Concomitantly, LTJ1/LTJ48 ameliorated hepatic and renal dysfunction, as evidenced: Serum creatinine (CRE) was reduced by 40.6% (LTJ1, *p* < 0.01) and 47.9% (LTJ48, *p* < 0.001), blood urea nitrogen (BUN) was lowered by 21.1% (LTJ1, *p* < 0.05) and 30.0% (LTJ48, *p* < 0.01), and aspartate aminotransferase (AST) decreased by 17.4% (LTJ1, *p* < 0.05) and 25.9% (LTJ48, *p* < 0.01). However, no significant changes in alanine aminotransferase (ALT) were observed (*p* > 0.05) (Figure 4B–E). Mechanistically, while neither strain inhibited xanthine oxidase (XOD) activity, both significantly suppressed adenosine deaminase (ADA) activity (LTJ1: 40.0%, *p* < 0.001; LTJ48: 47.5% reduction, *p* < 0.001) (Figure 4F,G), aligning with cellular findings. These findings align with in vitro results, highlighting ADA suppression—rather than XOD inhibition—as the primary mechanism underpinning the anti-HUA effects of both strains.

### 3.5. L. plantarum LTJ1 and LTJ48 Modulate UA Transporters in the Liver and Kidney Tissue of HUA Mice

**Figure 5:**
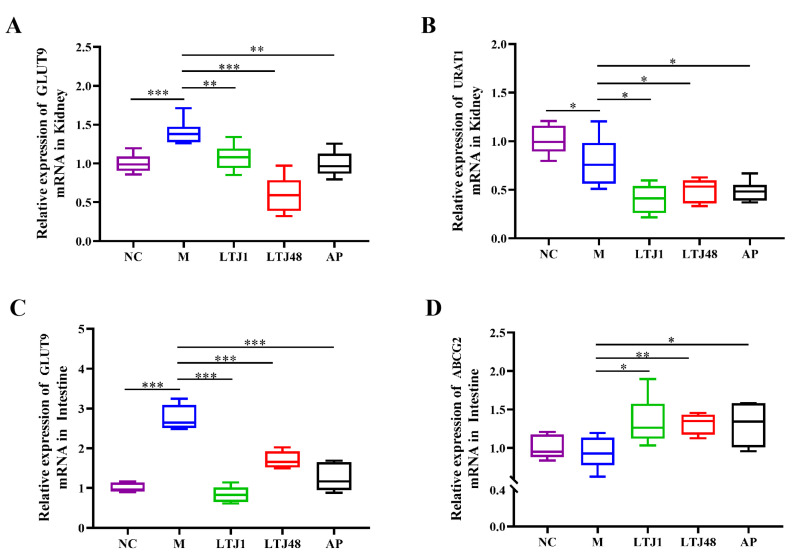
Multi-target regulation of uric acid transporters by *L. plantarum* LTJ1 and LTJ48 in HUA mice. (**A**) Renal GLUT9 expression; (**B**) renal URAT1 expression; (**C**) intestinal GLUT9 expression; and (**D**) intestinal ABCG2 expression. NC was the control group. M was the HUA model group. AP was the group treated with the positive drug allopurinol. The LTJ1 group was given live *L. plantarum* LTJ1. The LTJ48 group was given inactivate *L. plantarum* LTJ48. Data represent mean ± SD (n = 6/group). Statistical significance vs. HUA model group: * *p* < 0.05, ** *p* < 0.01, *** *p* < 0.001.

To elucidate the molecular mechanisms underlying UA reduction, we analyzed key urate transporters: renal URAT1, intestinal ATP-binding cassette sub-family G member 2 (ABCG2), and dual-localized glucose transporter 9 (GLUT9). Both strains significantly suppressed UA reabsorption by downregulating GLUT9 (LTJ1, 22.9% reduction, *p* < 0.001 and LTJ48, 57.0% reduction, *p* < 0.001) and URAT1 (LTJ1, 48.4% reduction, *p* < 0.001 and LTJ48, 36.7% reduction, *p* < 0.001) expression compared to the HUA model group in the kidney (Figure 5A,B). Concurrent inhibition of GLUT9 (LTJ1, 69.6% reduction, *p* < 0.001 and LTJ48, 38.1% reduction, *p* < 0.001) and was accompanied by a 1.4-fold upregulation (LTJ1, *p* < 0.05 and LTJ48, *p* < 0.01) of the excretion transporter ABCG2 (Figure 5C,D) in the small intestine. This dual-tissue, multi-transporter modulation—inhibiting reabsorption (URAT1/GLUT9) while enhancing excretion (ABCG2)—demonstrates a novel mechanism for UA homeostasis, distinct from single-target pharmacological approaches.

### 3.6. L. plantarum LTJ1 and LTJ48 Restore Gut Microbiota Homeostasis in HUA Mice

**Figure 6:**
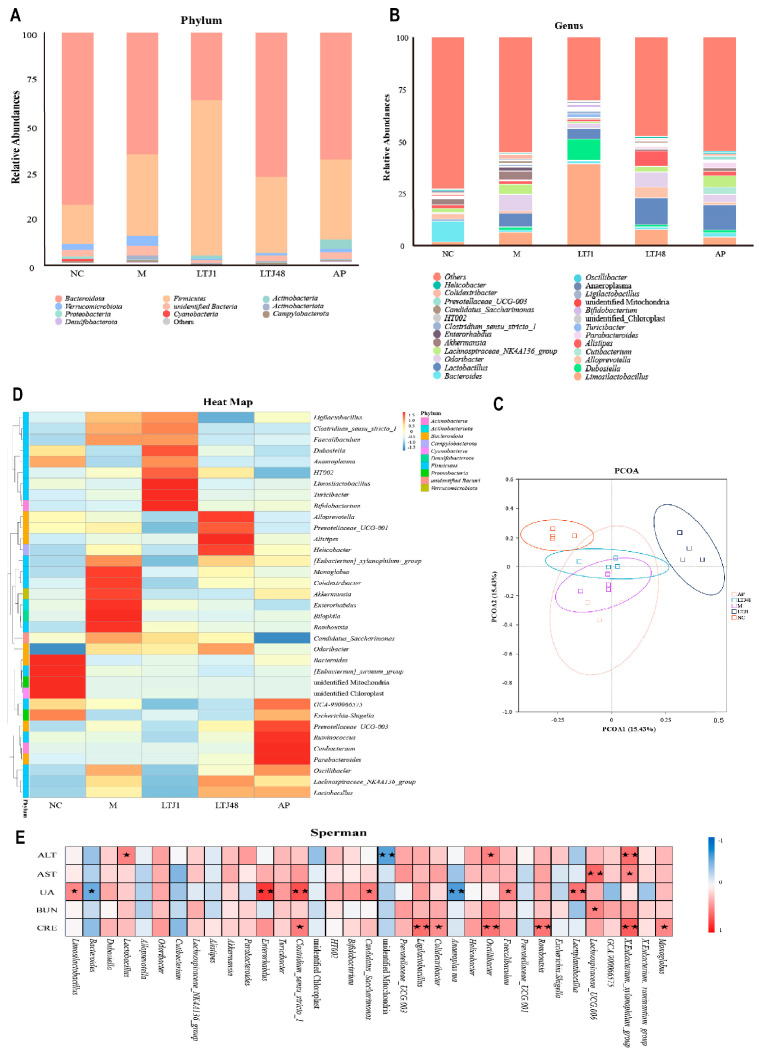
Functional and structural modulation of gut microbiota by *L. plantarum* LTJ1 and LTJ48 in HUA models. (**A**) Phylum-level microbiota composition among different groups; (**B**) genus-level abundance of dominant taxa; (**C**) principal coordinate analysis (PCoA) based on Bray−Curtis di−similarity; and (**D**) heatmap of taxonomic clustering patterns. NC was the control group. M was the HUA model group. (**E**) Spearman correlations between biomarkers (ALT, AST, UA, BUN, and CRE) and microbiota genera. AP was the group treated with the positive drug allopurinol. The LTJ1 group was given live *L. plantarum* LTJ1. The LTJ48 group was given inactivate *L. plantarum* LTJ48. Data represent mean ± SD (n = 6/group). Statistical significance vs. HUA model group: * *p* < 0.05, ** *p* < 0.01.

Through 16S rRNA sequencing analysis, we comprehensively explored the dynamic regulation of intestinal microbiota in HUA mice by *L. plantarum* LTJ1 and LTJ48 (Figure 6). At the phylum level, the dominant taxa were *Bacteroidetes*, *Firmicutes*, *Actinobacteria*, and *Verrucomicrobia* (Figure 6A). Comparative analysis revealed that the relative abundance of *Bacteroidetes* decreased in the model group, while *Firmicutes* and *Verrucomicrobia* increased. Treatment with LTJ1 further reduced *Bacteroidetes* and *Verrucomicrobia*, accompanied by a significant increase in *Firmicutes* and *Actinobacteria* compared to the model group. LTJ48 interventions, conversely, reduced *Verrucomicrobia* abundance while enhancing *Bacteroidetes* compared to the model group. At the genus level, *Bacteroides* dominated in the control group, with limited genus diversity (Figure 6B). In the model group, the relative abundance of *Lactobacillus*, *Odoribacter*, and *Lachnospiraceae* exceeded that of the control group. The LTJ1 group exhibited higher abundance of *Firmicutes*, particularly *Limosilactobacillus* and *Dubosiella*, whereas the LTJ48 group showed reduced *Lachnospiraceae* but increased *Lactobacillus* and Alloprevotella, distinguishing it from the control, model, and positive drug groups. The AP group displayed decreased *Oscillibacter* and *Lachnospiraceae* alongside elevated *Lactobacillus* compared to the model group.

Principal coordinates analysis (PCoA) (Figure 6C) highlighted distinct clustering between the control and model groups, reflecting dietary impacts on microbiota composition. Notably, the LTJ1 group formed a unique cluster, differing from the model, LTJ48, and drug groups, illustrating strain-specific microbial restructuring. Heatmap analysis (Figure 6D) further delineated taxon correlations. The NC group was associated with *Bacteroides*, while the model group was enriched in *Romboutsia*, *Monoglobus*, *Colidextribacter*, *Enterorhabdus*, and *Bilophilla*; the LTJ1 group was linked to *Dubosiella*, *Limosilactobacillus*, *Ligilactobacillus*, *Turicibacter*, *Bifidobacterium*, and *Clostridium*; the LTJ48 group was enriched in *Alloprevotella*, *Alistipes*, *Helicobacter*, and *Prevotellaceae*; and the AP group was correlated with *Ruminococcus*, *Oscillibacter*, and *Cutibacteroides.* Spearman correlation analysis (Figure 6E) identified significant associations. UA levels were positively correlated with *L. plantarum*, *Lactobacillus*, and *Clostridium*, but negatively correlated with *Bacteroides* and *Anaeroplasma*. Collectively, these findings demonstrate that LTJ1 and LTJ48 interventions induce significant intestinal microbiota remodeling in HUA mice.

## 4. Discussion

Probiotic supplementation offers a safer and more accessible alternative to conventional pharmaceuticals for HUA management [22,26]. While *Lactobacillus* strains are recognized for their therapeutic potential in metabolic disorders such as HUA and inflammation [27,28], the mechanisms underlying their UA-lowering effects—beyond direct purine degradation—remain poorly understood. This study pioneers a holistic investigation of *L. plantarum* LTJ1/LTJ48, shifting focus from isolated purine metabolism to systemic impacts on hepatic/renal function and gut microbiota ecology.

Previous studies have identified *Lactobacillus* strains with variable purine and UA degradation capacities, highlighting their strain-specific metabolic potential [29,30,31,32]. Building on this foundation, we screened and characterized *L. plantarum* LTJ1 and LTJ48, which achieved near-complete nucleoside degradation and reduced total purines by 35.2% and 42.2%, respectively, within 8 h—surpassing the performance of *Lactobacillus reuteri* TSR332 (90% inosine assimilation in 30 min) [29] and *Limosilactobacillus fermentum* JL-3 (40.9% UA reduction over 24 h) [30]. Critically, cross-study comparisons of degradation efficacy are confounded by methodological heterogeneity, including variations in substrate composition, incubation duration, and analytical protocols.

In the study of purine metabolism, the impact of the substances on ADA enzyme activity emerged as a key consideration. Expanding beyond nucleoside degradation, we evaluated LTJ1/LTJ48 metabolites in a hyperuricemic HepG2 cell model, observing a 24 h reduction in UA levels accompanied by downregulated PNP and ADA gene expression and ADA activity. These findings reveal a multi-target mechanism extending beyond purine catabolism. In vivo, a 21-day intervention with LTJ1/LTJ48 in HUA mice reduced serum UA levels. Parallel research endeavors yielded similar findings: *Pediococcus acidilactici* GQ01 and *Priestia megaterium* ASC-1 exhibited reductions of 40.87% [23] and 67.24% [33], respectively, in comparison to their corresponding model groups. Elevated CRE, BUN, and AST—markers of renal and hepatic dysfunction [34,35,36]—were also ameliorated by LTJ1/LTJ48. Notably, while high-purine diets increased XOD activity, LTJ1/LTJ48 selectively suppressed ADA without affecting XOD, consistent with cellular findings. This concordance between in vitro and in vivo results underscores the robustness of their anti-HUA effects.

Impaired UA excretion, driven by excessive renal reabsorption (~90% of filtered UA), is a central mechanism in HUA pathogenesis. This process is regulated by UA transporters: reabsorption mediators GLUT9 and URAT1, and the excretion pump ABCG2 [37,38]. In HUA, dysregulation of these transporters—characterized by upregulated GLUT9/URAT1 and downregulated ABCG2—promotes UA retention [39,40]. Our study demonstrates that LTJ1/LTJ48 restores UA homeostasis by downregulating GLUT9/URAT1 and upregulating ABCG2 in the kidney or small intestine. This dual-action mechanism simultaneously reduces UA reabsorption and enhances excretion, offering a novel therapeutic strategy for HUA management.

Under high-purine dietary conditions, the model group exhibited a significant microbial dysbiosis characterized by elevated abundances of *Odoribacter*, *Lachnospiraceae*, and *Bacteroides* compared to the NC group—a pattern consistent with HUA-associated enrichment of opportunistic pathogens. Notably, *Lactobacillus* proliferation observed in untreated HUA models may reflect compensatory responses to dietary stress rather than therapeutic effects. In contrast, targeted supplementation with *L. plantarum* strains LTJ1 and LTJ48 induced substantial structural reorganization of gut microbiota, driving differential probiotic enrichment. The LTJ1 intervention uniquely elevated *Limosilactobacillus* (relieves HUA [41]), *Dubosiella* (intestinal anti-inflammatory modulator [42]), *Faecalibacterium* (typically depleted in HUA patients [43]), and *Bifidobacterium*, accompanied by *Firmicutes* dominance and distinct PCoA clustering. Parallel effects emerged in LTJ48-treated mice showing reduced *Lachnospiraceae* and *Enterobacter* alongside increased *Alistipes* (cholesterol regulator [44]) and *Alloprevotella* (SCFA producer countering metabolic inflammation [45]). Crucially, both probiotics suppressed disease-exacerbating taxa while amplifying commensals with documented roles in barrier protection and UA metabolism regulation.

This study provides the necessary theoretical support for the commercial development of new probiotics. New probiotics can be used to develop new commercial products for improving hyperuricemia, such as functional foods and beverages, including probiotic yogurt/fermented milk, probiotic solid beverages/granules, and probiotic energy bars/snacks; dietary supplements, including probiotic capsules for high uric acid and intestinal-uric acid metabolism joint regulation products; and medical-grade products, which are mainly auxiliary treatment preparations used in conjunction with low-purine diets.

## 5. Conclusions

In summary, this investigation elucidates the dual probiotic potential of *L. plantarum* strains LTJ1 and LTJ48 in mitigating HUA through nucleoside and purine degradation. Systematic evaluation across cellular and murine HUA models revealed their significant urate-lowering efficacy coupled with modulatory effects on gut microbiota composition and metabolic functionality. Notably, these strains offer a targeted microbial therapeutic approach distinct from conventional HUA interventions, demonstrating direct enzymatic catabolism of purine precursors, restorative microbiota remodeling, and synergistic reduction of serum UA biomarkers. This evidence positions microbiome-based modulation as an innovative prophylactic strategy for chronic HUA management and its associated comorbidities.

## Figures and Tables

**Table 1:** Sequences of RT-qPCR specific primer.

Target Gene	Forward Primer	Reverse Primer
hADA	GCCTTCGACAAGCCCAAAGTA	CTCTGCTGTGTTAGCTGGGAG
hPNP	CTAAGCACCGACCTCAAGTTG	TGGGGATTTCACCGTAGTCAAAGA
hGAPDH	CTCCTGCACCACCAACTG	CAGTGATGGCATGGACTGTGGTC
mGLUT9	GTGAAAAGAACTCCGCAGAAACCA	AGGAAGGAGGACCCGAAGGCTC
mABCG2	ATGTCTTCCAGTAATGACCACGTGTTAG	GATGAAAACTCAACACATCTCCTTC
mURAT1	TCCTGAACTCCTGGACCGAGTG	AGTTCTCCAGCATGTTCTGA
mGAPDH	ATGGTGAAGGTCGGTGTGAACGG	TGGAACATGTAGACCATGTAGTTGAGG

## Data Availability

The data presented in this study are available within the article.

## Abbreviations

The following abbreviations are used in this manuscript.

HUA	Hyperuricemia
XOD	Xanthine oxidase
URAT1	Urate transporter 1
ADA	Adenosine deaminase
CRE	Serum creatinine
BUN	Blood urea nitrogen
AST	Aspartate aminotransferase
ALT	Alanine aminotransferase
ABCG2	ATP-binding cassette sub-family G member 2
GLUT9	Glucose transporter 9
